# Revisiting Eye Movement Desensitization and Reprocessing Therapy for Post-traumatic Stress Disorder: A Systematic Review and Discussion of the American Psychological Association’s 2017 Recommendations

**DOI:** 10.7759/cureus.58767

**Published:** 2024-04-22

**Authors:** Sasha Vereecken, German Corso

**Affiliations:** 1 Department of Medicine, Saint James School of Medicine, The Quarter, AIA; 2 Department of Child and Adolescent Psychiatry, Tropical Texas Behavioral Health, Harlingen, USA

**Keywords:** cognitive behaviour therapy, hypnosis, psychotherapy, eye movement desensitization and reprocessing, post traumatic stress disorder (ptsd)

## Abstract

This literature review evaluates the efficacy and clinical applications of eye movement desensitization and reprocessing (EMDR) therapy for post-traumatic stress disorder (PTSD). The review highlights the effectiveness of EMDR in reducing PTSD symptoms and explores variations in treatment protocols, populations studied, and outcome measures. We conducted systematic searches of multiple databases, supplemented with manual searches and reference list exploration. The inclusion criteria focused on English-language studies published between January 2000 and June 2023, with a specific emphasis on adult psychiatric patients with PTSD receiving EMDR treatment. The review utilized Preferred Reporting Items for Systematic Reviews and Meta-Analyses (PRISMA) guidelines for narrative literature reviews. Out of 867 identified studies, 16 met the eligibility criteria. Most studies found that EMDR was superior in relieving PTSD when compared to controls. Eleven of the 16 selected studies demonstrated improvement in PTSD symptoms. An additional three studies noted an improvement in PTSD symptoms when compared to their waitlist control counterparts. One study found EMDR superior in combating depressive symptoms when compared to rapid eye movement desensitization. EMDR therapy is an appropriate treatment for PTSD. Although some studies compared to waitlist controls, and others have a small number of participants, the data supports the use of EMDR for PTSD. Future studies are needed to continue to better understand the mechanism and application in different populations.

## Introduction and background

Post-traumatic stress disorder (PTSD) is a prevalent and debilitating mental health condition that arises following exposure to traumatic events. It affects individuals of all ages and backgrounds and can have a profound impact on their overall well-being and quality of life. According to the WHO, 22% of individuals living in an area affected by conflict are estimated to have depression, anxiety, PTSD, bipolar disorder, or schizophrenia [1].

Various therapeutic interventions have been developed over the years to address PTSD, including Eye Movement Desensitization and Reprocessing (EMDR) by Shapiro F in the 1980s [2]. EMDR is a psychotherapy technique that involves guided eye movements to process traumatic memories and alleviate PTSD symptoms. EMDR remains one of the main recommended psychological interventions for the treatment of trauma by multiple national and international governing bodies [3-5]. While EMDR has shown promise in some studies, there remains a gap in knowledge regarding its overall effectiveness and its comparison with other standard treatments for PTSD.

Our study aims to investigate the effect of EMDR in the treatment of PTSD. Specifically, the research will focus on comparing the efficacy of EMDR to other standard treatments for PTSD, such as cognitive-behavioral therapy and pharmacotherapy. By addressing this research question, we aim to gain further support for its use from the American Psychological Association (APA).

The implications of this research are twofold. First, by assessing the effectiveness of EMDR as a treatment for PTSD, we hope to establish that EMDR is comparable to existing treatments. Second, it would provide evidence of its usefulness as an alternative therapeutic option, giving clinicians and patients more choices for customized treatment plans based on individual needs and preferences. The target population for this study includes adults diagnosed with PTSD, spanning various demographics and trauma types. By pooling and reviewing studies on this topic, we can gather a comprehensive understanding of EMDR’s effectiveness and identify potential areas for further research and improvement in the treatment of PTSD.

## Review

Several studies have explored the use of EMDR as a therapeutic intervention for PTSD. A 2007 meta-analysis conducted by Bisson J and Andrew M reviewed the effectiveness of various psychological treatments for PTSD, including EMDR, which was found to be associated with significant reductions in PTSD symptoms compared to controls [6]. EMDR therapy is recognized for incorporating elements of both cognitive behavioral therapy (CBT) and exposure therapy, utilizing eye movements during the exposure phase. The patient is asked to track the therapist’s fingers back and forth with their eyes as they visualize the traumatic event.

Methodology

Search Strategy

**Figure 1:**
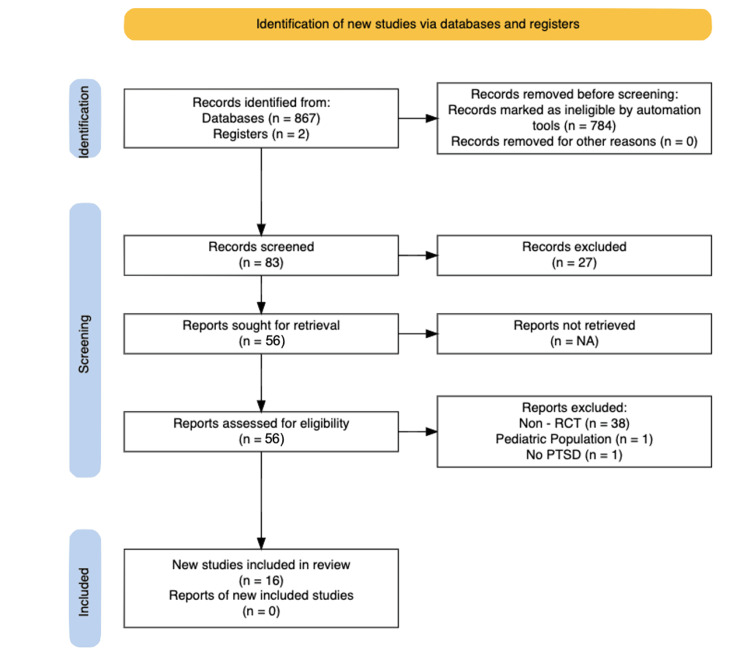
PRISMA Search strategy flow diagram

A systematic search of PubMed, Medline, Embase, Web of Science, and the Grey Literature databases was conducted to identify all studies published in English that included both EMDR and PTSD. Unpublished studies were excluded as there was not sufficient access to data to ensure they met inclusion criteria. Articles from January 2000 to June 2023 were collected. This systematic literature review was performed with reference to the Preferred Reporting Items for Systematic Reviews and Meta-Analyses (PRISMA) guidelines. The following Medical Subject Headings (MeSH) search terms were used: 'Eye Movement Desensitization and Reprocessing Therapy' and 'PTSD (Post-Traumatic Stress Disorder)'. We limited results to full-text availability, and duplicate records were removed. Reference lists and related articles from retrieved documents were also searched. Computer searches were supplemented with a manual search. An independent screening of all citations in abstracts was selected by the search strategy to identify potentially eligible studies. Figure 1 shows a flow diagram detailing the main steps of the search phase.

Eligibility Criteria

The study eligibility criteria included English-language publications from January 2000 to June 2023, focusing on adult psychiatric patients diagnosed with PTSD who underwent EMDR therapy as part of their treatment plan. The selected studies needed to be randomized controlled trials published in peer-reviewed journals. By employing these criteria, the review aimed to gather a relevant and reliable pool of studies to assess the effectiveness of EMDR as a therapeutic intervention for PTSD in the specified population and timeframe.

Exclusion Criteria

The study exclusion criteria were designed to ensure the focus and relevance of the review. Studies involving pediatric populations were excluded from consideration, as the review specifically targeted adult psychiatric patients with PTSD. Additionally, unpublished studies were not included to maintain the integrity of the research and ensure the availability of sufficient data for analysis. By excluding studies that did not meet these criteria, our review aimed to concentrate on a well-defined and homogeneous group of research, thereby enhancing the reliability and applicability of its findings to the adult population seeking EMDR therapy for PTSD during the specified period.

Data Extraction

One reviewer independently retrieved the following data from articles: first author, title, year, study type, population, number of participants, interventions, and any outcomes of interest. Assessment of articles was done using the Grading of Recommendations Assessment, Development, and Evaluation (GRADE) framework [7]. A second reviewer checked for any duplicates and discussed with the first reviewer any disagreements regarding eligibility.

Results

Through our database search, a total of 865 studies were identified from the existing literature. After further screening and duplication checks, a total of 83 were selected. Of these, 57 met our inclusion criteria. Studies were then reviewed and categorized based on various factors. A total of 38 were further excluded for not meeting the study type, one for not meeting the population type, and one for not discussing PTSD. Eleven of the 16 studies demonstrated a statistically significant improvement in PTSD symptoms when compared to controls. An additional three studies noted an improvement in PTSD symptoms when compared to their waitlist control counterparts, demonstrating the continued benefit of treatment in settings where no other treatments are available or accessible (e.g., in war-torn regions). Ahmadi K et al. demonstrated that compared to rapid eye movement (REM) desensitization, EMDR was only superior in combating depressive symptoms [8].

EMDR Efficacy

It is noteworthy that of the 16 selected studies shown in Table 1, more than two-thirds displayed statistically significant results in improving PTSD symptoms. EMDR is highly efficacious as demonstrated by the supporting studies. Treatment response rates showed a marked improvement, as seen in Acarturk C et al., where at the one-month post-assessment, control participants were 23 times more likely to be diagnosed with PTSD compared to the EMDR group [9]. Although van den Berg DP et al. demonstrated that EMDR was superior to waitlist control groups, the study proved that the use of EMDR does not result in any adverse effects [10]. This provides continued support for the proven safety and tolerability of EMDR when compared to other interventions. Specific Quality of Life (QoL) measures were not utilized in any of the selected studies; however, this would be an important aspect for future research. It can now be posited that an improvement in PTSD symptomatology (flashbacks, intrusive thoughts, nightmares, intense distress, physical sensations, etc.) would lead to marked improvements in other facets of living.

**Table 1:** Comparing EMDR efficacy in PTSD. EMDR: Eye movement desensitization and reprocessing; PTSD: Post-traumatic stress disorder; CBT: Cognitive-behavioral therapy; RCT: Randomised controlled trial.

Last name, year	Study Design	N	Interventions	Outcomes Measured	Results
Acarturk C et al., (2015) [11]	RCT	29	EMDR	PTSD symptoms	Improvement
Acarturk C et al., (2016) [9]	RCT	98	EMDR	Efficacy in treating PTSD and depression among Syrian refugees	Improvement
Ahmadi K et al., (2015) [8]	RCT	33	REM Desensitization	PTSD	EMDR has superior effect than REM Desensitization in Depression symptoms alone
Carletto S et al., (2016) [12]	RCT	22	EMDR, Relaxation Therapy	PTSD in patients with Multiple Sclerosis	Improvement
de Bont PA et al., (2016) [13]	RCT	155	EMDR, Prolonged Exposure Therapy	Comparison of the effects of prolonged exposure and EMDR on symptoms of psychosis, depression, and social functioning in patients with chronic psychotic disorders	Superior to Waitlist
Högberg G et al., (2007) [14]	RCT	24	EMDR	Chronic PTSD in transportation workers	Improvement
Lenferink LI et al., (2020) [15]	RCT	39	EMDR, Cognitive Therapy	Efficacy of EMDR and cognitive therapy in reducing psychopathology in bereaved individuals after the MH17 plane crash	Not statistically significant
Nijdam MJ et al., (2012) [16]	RCT	140	EMDR, Brief Eclectic Psychotherapy	Comparison of brief eclectic psychotherapy and EMDR in treating PTSD	Improvement
Perri RL et al., (2021) [17]	RCT	42	EMDR, CBT	Efficacy of internet-based EMDR and CBT for ongoing trauma during COVID-19	Improvement
Rothbaum BO et al., (2005) [18]	RCT	60	EMDR, Prolonged Exposure Therapy	Comparison of prolonged exposure and EMDR in treating PTSD in rape victims	Improvement
Sack M et al., (2016) [19]	RCT	139	EMDR	Comparison of dual attention, eye movements, and exposure during EMDR for PTSD	Improvement
Servan-Schreiber D et al., (2006) [20]	RCT	21	EMDR	Comparison of different stimulation types in EMDR treatment for PTSD	Improvement
Ter Heide FJ et al., (2016) [21]	RCT	72	EMDR, Stabilization as Usual	Refugees with PTSD	Superior to Waitlist
van den Berg D et al., (2015) [10]	RCT	155	EMDR, Prolonged Exposure Therapy	Comparison of prolonged exposure, EMDR, and waiting list for PTSD in patients with a psychotic disorder	Superior to Waitlist
van der Kolk BA et al., (2007) [22]	RCT	88	EMDR, Fluoxetine, Pill Placebo	PTSD	Improvement
Wippich A et al., (2023 [23]	RCT	268	EMDR	Efficacy of EMDR in conflict-affected areas	Improvement

Discussion

Accessibility

In many countries experiencing ongoing political unrest and war, access to mental health support can seem impossible. CBT is one of the common treatment options for PTSD; however, it is not always user-friendly. It requires personal time outside therapy sessions to complete homework, which necessitates access to a place to view these documents and space to store them. In settings such as refugee camps or shelters, access to a private space for completing and storing such documents is limited, posing a major barrier to treatment. EMDR remains a glimmer of hope for those who have experienced trauma, as it requires no additional homework and is highly accessible. EMDR is considered the most cost-effective treatment for PTSD in adults [13, 24]. The simplicity of EMDR makes it feasible for trained therapists to provide the treatment even in resource-limited environments, where traditional talk therapies might be difficult to implement and may be frowned upon.

Furthermore, EMDR's structured protocol allows it to be tailored to various cultural contexts, enabling therapists to integrate local beliefs and practices while maintaining its core principles. As discussed in Acarturk C et al., “Majnun” is a term used in Syria to label and outcast others from the community as insane [9]. In this study, treatment was discreetly provided in the same building as a daycare center. Another study by Perri RL et al. successfully provided EMDR virtually during the COVID-19 pandemic [17]. Capitalizing on its accessibility and adaptability, EMDR therapy holds the promise of reaching individuals scarred by the psychological wounds of war, offering them a path to healing and recovery during adversity.

Waitlist Controls

The approach of using waitlist controls is common in the field of psychology. Essentially, a waitlist control is placed on a “waiting list” to receive the appropriate treatment while the treatment group receives the intervention first. The comparison of a waitlist control allows researchers to determine if the results are due to the intervention or extraneous factors. The controls are deemed ethically responsible as they eventually receive the treatment and standard of care. Researchers must carefully weigh the risks of negative implications when using waitlist controls. Ethical considerations, confounding variables, and the appropriateness of delaying treatment all need to be considered to ensure that the benefits of using waitlist controls outweigh the drawbacks in each study.

Proposed Mechanism

EMDR operates on a comprehensive mechanism that aims to alleviate the distressing effects of traumatic memories and experiences. Central to this approach is the Adaptive Information Processing (AIP) model, which posits that the brain's natural capacity to process and integrate information can be hindered by traumatic events, leading to the persistence of distressing emotions and sensations. The key aspect of EMDR is the use of bilateral stimulation, which can be achieved through back-and-forth eye movements, tapping, or auditory cues. The exact mechanism by which bilateral stimulation aids EMDR remains a topic of debate and requires further research. It is believed that the core symptomatology in PTSD can be seen as fear learning, in accordance with Pavlov’s classical description: “The basic condition for a conditioned reflex is a single or repeated coincidence in time of indifferent stimuli with unconditioned reflexes” [14]. This conditioned response can be altered through counterconditioning, defined by Wolpe as: “If a response antagonistic to anxiety can be made to occur in the presence of anxiety-evoking stimuli so that it is accompanied by a complete or partial suppression of the anxiety responses, the bond between these stimuli and the anxiety responses will be weakened” [25]. Studies in mice have shown that EMDR and the effect of alternating bilateral sensory stimulation can shift the balance between competing brain circuits, favoring neural pathways that facilitate fear extinction and inhibit pathways that perpetuate fear [26].

APA Recommendation Review

As of the publication of this document, the 2017 Clinical Practice Guideline for the Treatment of PTSD by the APA has listed the use of EMDR as a “Conditionally Recommended” treatment option [5]. With the support of the literature described in our systematic review, it necessitates a reconsideration for reclassification into the “Strongly Recommended” treatment options in future practice guidelines. Currently, CBT, CPT, Cognitive Therapy, and Prolonged Exposure are within the “Strongly Recommended” category. It is noteworthy that multiple studies have found EMDR to be superior to, or at least as effective as, CBT and Prolonged Exposure in reducing PTSD symptoms [10, 13, 15, 17, 18]. Further research and review are recommended with support from the APA.

Strengths and limitations 

Performing a systematic review offers several strengths that contribute to its significance in research and evidence-based decision-making. Firstly, a systematic review follows a well-defined and rigorous methodology, ensuring transparency and minimizing bias. By comprehensively searching and evaluating all available relevant studies, we were able to provide an overview of the existing evidence and an unbiased summary of the effectiveness and safety of EMDR therapy in treating PTSD. Our systematic approach enriches the integrity of the findings and supports evidence-based decision-making in clinical practice. We hope our review will add to the current body of research, serving as a formidable tool for making informed decisions in healthcare, policy, and various other fields. Moreover, systematic reviews enable the identification of trends, inconsistencies, and gaps in the literature, thus guiding future research directions.

The limitations of our review must also be acknowledged. The process is time-consuming and resource-intensive, requiring meticulous planning, expertise, and a substantial commitment of time. The quality of a systematic review heavily depends on the quality of the included studies, which might vary widely in terms of methodology, reporting, and biases. Populations varied across nations from Syria to the Netherlands and included digital mediums among many others. The experiences with trauma also varied, with some populations fleeing as refugees and others working in transportation. Selective publication and reporting bias can also affect the results, as negative or inconclusive findings may be less likely to be published. Despite these limitations, a well-executed systematic review remains an invaluable tool for integrating and appraising existing knowledge.

## Conclusions

EMDR has been a therapeutic approach for treating PTSD and other trauma-related conditions for decades. Its efficacy lies in aiding individuals in processing distressing memories and associated emotions. By diminishing their emotional impact, EMDR facilitates adaptive resolution for patients. The wealth of studies and clinical trials investigating the effectiveness of EMDR in addressing PTSD consistently underscores its value as a treatment option, as demonstrated in our systematic review. Beyond its capacity to alleviate symptoms, EMDR offers promise in promoting long-term healing and recovery for those grappling with the aftermath of trauma. Moreover, its adaptable nature lends itself well to telehealth platforms, making it accessible to individuals who may face barriers to in-person therapy. The integration of EMDR into telehealth services expands the reach of mental health care, particularly in underserved communities or for individuals with mobility constraints. This underscores the importance of EMDR not only as an effective treatment modality but also as a means of enhancing accessibility and equity in mental health services.
